# Receipt of Industry Payments and Surgeons’ Adoption of Robotic-Assisted Surgery

**DOI:** 10.1001/jamanetworkopen.2026.3885

**Published:** 2026-03-30

**Authors:** Wei San Loh, Edward C. Norton, Jyothi Thumma, Justin B. Dimick, Kyle H. Sheetz

**Affiliations:** 1 Center for Healthcare Outcomes and Policy, University of Michigan, Ann Arbor; 2 Department of Health Management and Policy, University of Michigan, Ann Arbor; 3 Department of Economics, University of Michigan, Ann Arbor; 4 Department of Surgery, University of Michigan, Ann Arbor

## Abstract

**Importance:**

The use of robotic-assisted surgery has increased rapidly despite limited evidence of superior outcomes over more established surgical approaches such as laparoscopy.

**Objective:**

To evaluate whether and to what extent surgeons’ financial relationships with industry are associated with the use of robotic-assisted surgery.

**Design, Setting, and Participants:**

In this cohort study, fee-for-service Medicare claims (January 1, 2011, to December 31, 2021) for patients undergoing 1 of 4 common surgical operations (bariatric surgery, cholecystectomy, colectomy, or ventral hernia repair) were linked to surgeon-level Open Payments data on receipt of industry payments from a large robotic surgical device company. Data were analyzed from April to August 2025.

**Exposure:**

Receipt of a direct industry payment from a robotic-assisted surgical device company.

**Main Outcome and Measures:**

Each surgeon’s use of robotic-assisted surgery as a proportion of all surgeries performed by that surgeon. A staggered difference-in-differences (DID) approach was used to isolate the association of industry payments with the proportional use of robotic-assisted surgery among surgeons who received payment compared with control surgeons who never received a payment.

**Results:**

Among 20 313 surgeons (mean [SD] age, 50.7 [10.2] years; 86.2% male) performing 886 385 surgeries, 5933 (29.2%) received at least 1 industry payment. Receipt of an industry payment was associated with a significant increase in the proportional use of robotic-assisted surgery, with a DID estimate of 9.9 percentage points (pp) (95% CI, 9.30-10.6 pp). Results were consistent across discrete procedures (eg, DID estimate of 11.7 pp [95% CI, 9.4-13.9 pp] for bariatric surgery and 10.3 pp [95% CI, 9.4-11.4 pp] for ventral hernia repair). There was a significant dose-dependent response. For example, surgeons receiving less than $500 increased use of robotic-assisted surgery after payment from a mean of 1.5% (95% CI, 1.4%-1.6%) to 3.7% (95% CI, 3.5%-3.9%), compared with 0.4% (95% CI, 0.4%-0.5%) to 17.0% (95% CI, 16.7%-17.3%) among surgeons receiving more than $10 000.

**Conclusions and Relevance:**

In this cohort study, receipt of industry payments by surgeons was associated with increased use of robotic-assisted surgery compared with no receipt of payment, with a significant dose-dependent response. These results suggest that surgeon-industry financial relationships may be an important contributor to greater use of robotic-assisted surgery across the US.

## Key Points

**Question:**

Are industry payments associated with an increase in the use of robotic-assisted surgery for common procedures?

**Findings:**

In this cohort study of 20 313 US surgeons, receipt of an industry payment was associated with a significant increase in the proportional use of robotic-assisted surgery compared with surgeons who never received payment, with a significant dose-dependent response to higher payment amounts.

**Meaning:**

The findings suggest that surgeon-industry financial relationships may be an important contributor to greater use of robotic-assisted surgery in the US.

## Teaser

This cohort study of US surgeons assesses whether receipt of industry payments from a robotic-assisted device company is associated with increased use of robotic-assisted surgery.

## Introduction

Over the past decade, robotic-assisted surgery has diffused rapidly across a wide range of clinical domains despite limited evidence of superior outcomes. For example, robotic-assisted inguinal hernia repairs have increased 40-fold, reflecting nearly one-third of all cases, even though a large randomized clinical trial showed similar clinical outcomes (eg, wound complications), patient-reported quality of life, and surgeon ergonomics compared with a traditional laparoscopic approach.^1,2^ Nonclinical factors may be more important in explaining trends toward greater adoption of robotic-assisted surgeries. Patients undergoing urologic or gynecologic surgeries are 2 to 5 times more likely to have robotic-assisted surgery in competitive vs noncompetitive hospital markets.^3,4^

Whether financial incentives or direct payments to physicians from industry also contribute to the rapid adoption of robotic-assisted surgery remains unclear. A recent study in New York State reported a 150% increase in industry payments to general surgeons between 2015 and 2020, coinciding with a 182% increase in robotic-assisted surgeries.^5^ Beyond surgery, there is significant evidence from the pharmaceutical industry that payments to physicians are associated with increased prescribing, with clear temporal and dose-dependent responses to payments.^6,7,8,9,10^ Coupling this with evidence that 40% to 90% of physicians receiving payments from a group of first-in-class agents also receive payments from their direct competitors indicates that these financial relationships are both common and strategic.^11^

We linked national Medicare claims to publicly available data to examine whether direct payments to surgeons from a large robotic-assisted surgical device company were associated with changes in use of this technology. We used a staggered difference-in-differences (DID) approach to investigate associations between industry payments and the use of robotic-assisted surgery among surgeons who had ever received a payment compared with surgeons who had not.

## Methods

### Data Sources and Study Population

This cohort study was designed to isolate the association between industry payments and surgeons’ subsequent use of robotic-assisted surgery among general surgical procedures experiencing the largest and most rapid adoption of the technology. We identified fee-for-service Medicare beneficiaries aged 18 years or older who underwent bariatric surgery, cholecystectomy, colectomy, or ventral hernia repair from January 1, 2011, through December 31, 2021, using 100% Medicare Provider Analysis and Review inpatient claims. Procedures were identified using appropriate *International Classification of Diseases, Ninth Revision (ICD-9)*, *International Statistical Classification of Diseases and Related Health Problems, Tenth Revision (ICD-10)*, and *Current Procedural Terminology (CPT)* procedure codes^12,13,14,15^ (eTable 1 in Supplement 1). These surgical procedures were selected because they represent common and rapidly growing applications of robotic-assisted surgeries. This study was deemed exempt from review and informed consent by the University of Michigan institutional review board due to the use of retrospective deidentified and publicly available data. We followed the Strengthening the Reporting of Observational Studies in Epidemiology (STROBE) reporting guideline for cohort studies.

We identified each primary surgeon’s National Provider Identifier (NPI) using Medicare Part B claims and linked it to publicly available data on industry relationships with the Centers for Medicare & Medicaid Services’ Open Payments database. We also linked the NPIs to the American Medical Association’s Physician Professional Data master file to obtain additional data on surgeons’ age, demographics, and training. We focused on all financial transactions (eg, payments for educational activities, consulting, or travel) made by Intuitive Surgical, Inc, the world’s largest manufacturer of robotic-assisted surgical systems, to surgeons identified in the Medicare cohort.^16^ During the study period, this manufacturer had a near complete capture of the robotic-assisted surgery market. Furthermore, we linked claims to the American Hospital Association Annual Survey to obtain additional data on hospital characteristics such as bed size, region, and other services offered.

### Outcomes and Exposure

Our primary outcome was the annual proportion of robotic-assisted surgeries performed by each surgeon, defined as the number of robotic-assisted procedures divided by the total number of robotic-assisted, laparoscopic, and open surgeries performed by that surgeon. The primary exposure was defined as the receipt of any direct industry payment from Intuitive Surgical, Inc, to individual surgeons. Surgeons were classified as treated beginning the first year in which they received such a payment. Surgeons who did not receive any payments throughout the study period served as the control group.

### Statistical Analysis

We used a staggered DID approach to estimate changes in surgeons’ use of robotic-assisted surgery following the receipt of an industry payment from Intuitive Surgical, Inc. We defined the treatment year as the surgeon’s first payment year. To account for potential residual confounding and baseline differences between the treated and control groups, we performed entropy balancing separately at each event time, reweighting the control group to match the covariate distribution of the treated group.^17^ Entropy balancing is a reweighting method that makes the comparison group look more similar to the treated group with respect to observed characteristics without excluding observations. In our data, this was reflected by fewer differences in patient, hospital, and surgeon characteristics, with SDs near 0 after weighting (Table 1). We evaluated the parallel trends assumptions by assessing pretreatment differences in the slopes of proportional use of robotic-assisted surgeries between the treated and control groups (eTable 2 in Supplement 1).

**Table 1.** Surgeon and Patient Characteristics

Characteristic	Participants^a^					
	Before entropy balancing			After entropy balancing		
	Never received payment	Received payment	Standardized difference	Never received payment	Received payment	Standardized difference
**Surgeons**						
Total, No. (N = 20 313)	14 380	5933	NA	14 380	5933	NA
Age, mean (SD), y	53.0 (10.1)	47.2 (9.2)	−60.6	47.2 (9.5)	47.2 (9.2)	−0.1
Sex						
Female	1941 (13.5)	872 (14.7)	3.4	2114 (14.7)	872 (14.7)	0.0
Male	12 439 (86.5)	5061 (85.3)	−3.4	12 266 (85.3)	5061 (85.3)	0.0
Experience since medical school, mean (SD), y	26.0 (10.6)	20.1 (9.6)	−58.4	20.1 (9.9)	20.1 (9.6)	−0.1
**Patients**						
Total, No. (N = 886 385)	531 137	355 248	NA	531 137	355 248	NA
Age, mean (SD), y	73.0 (10.8)	72.4 (11.0)	−5.7	72.4 (10.9)	72.4 (11.0)	0.0
Sex						
Female	296 374 (55.8)	202 847 (57.1)	2.7	303 279 (57.1)	202 847 (57.1)	0.0
Male	234 763 (44.2)	152 401 (42.9)	−2.7	227 858 (42.9)	152 401 (42.9)	0.0
Race and ethnicity						
Asian	6374 (1.2)	3908 (1.1)	−1.3	6374 (1.2)	3908 (1.1)	−1.4
Black	44 084 (8.3)	32 328 (9.1)	2.8	45 147 (8.5)	32 328 (9.1)	2.1
Hispanic	11 154 (2.1)	7460 (2.1)	0.4	11 154 (2.1)	7460 (2.1)	−0.2
North American Native	4249 (0.8)	2131 (0.6)	−2.5	4780 (0.9)	2131 (0.6)	−3.2
White	457 840 (86.2)	304 803 (85.8)	−1.1	455 716 (85.8)	304 803 (85.8)	0.0
Other^b^	7436 (1.4)	4618 (1.3)	−0.9	7436 (1.4)	4618 (1.3)	−1.3
Elixhauser index, mean (SD)^c^	3.15 (2.0)	3.17 (1.9)	1.1	3.17 (2.0)	3.17 (1.9)	0.0
Procedure						
Bariatric surgery	18 556 (3.5)	29 255 (8.2)	20.3	24 918 (4.7)	29 255 (8.2)	15.2
Cholecystectomy	221 040 (41.6)	129 841 (36.5)	−10.4	230 383 (43.4)	129 841 (36.5)	−14.2
Colectomy	191 511 (36.1)	133 975 (37.7)	3.4	182 629 (34.5)	133 975 (37.7)	6.6
Hernia repair	100 030 (18.8)	62 177 (17.5)	−3.5	91 865 (17.3)	62 177 (17.5)	0.5
**Hospitals** ^d^						
For-profit status	62 143 (11.7)	52 577 (14.8)	9.0	78 608 (14.8)	52 577 (14.8)	0.0
Teaching hospital	111 539 (21.0)	69 629 (19.6)	−3.6	104 103 (19.6)	69 629 (19.6)	0.0
Beds, No.						
<200	190 147 (35.8)	92 364 (26.0)	−21.3	156 154 (29.4)	92 364 (26.0)	−7.4
200-349	125 348 (23.6)	94 851 (26.7)	7.3	121 630 (22.9)	94 851 (26.7)	8.8
350-499	80 202 (15.1)	68 563 (19.3)	11.4	88 700 (16.7)	68 563 (19.3)	6.9
≥500	135 971 (25.6)	99 469 (28.0)	5.3	164 652 (31.0)	99 469 (28.0)	−6.8
Region						
Midwest	126 411 (23.8)	79 220 (22.3)	−3.5	113 132 (21.3)	79 220 (22.3)	2.5
Northeast	108 352 (20.4)	54 353 (15.3)	−13.2	97 198 (18.3)	54 353 (15.3)	−7.8
South	205 019 (38.6)	160 217 (45.1)	13.1	224 671 (42.3)	160 217 (45.1)	5.7
West	91 355 (17.2)	61 103 (17.2)	0.1	96 136 (18.1)	61 103 (17.2)	−2.3
Urban	467 932 (88.1)	304 092 (85.6)	−7.3	454 653 (85.6)	304 092 (85.6)	0.0

Abbreviation: NA, not applicable.

^a^ Data are presented as number (percentage) of surgeons or patients unless otherwise indicated.

^b^ Other race is not broken down further in Medicare inpatient claims.

^c^ Calculated as the sum of 29 Elixhauser comorbidities (score range, 0-29, with higher scores indicating greater comorbidity burden).

^d^ Data reflect the patient distribution for each hospital characteristic.

We used weighted linear regression models with robust SEs clustered at the surgeon level. Models were adjusted for patient demographics (age, sex, race, and ethnicity) and comorbidity burden (sum of 29 Elixhauser comorbidities, ranging from 0 to 29); hospital characteristics (teaching status, ownership type, bed size category, urban location, and census region); surgeon characteristics (age, sex, and years of experience); and calendar-year fixed effects, which also helped account for COVID-19 pandemic–related disruptions during 2020 and 2021. Patients’ self-reported race and ethnicity were included in the analysis as a social construct to account for disparities; categories were Asian, Black, Hispanic, North American Native, White, and other race (not broken down further in Medicare inpatient claims). Because hospital robot ownership was unobserved, we adjusted for hospital characteristics and used entropy balancing to improve comparability between the treated and control groups. Marginal effects were computed to estimate the adjusted annual proportion of robotic-assisted surgery use.

We performed several sensitivity analyses to ensure the robustness of our primary findings. First, we tested our DID estimates in an unadjusted model. Next, we used the Callaway and Sant’Anna^18^ method and a flexible linear specification proposed by Deb et al^19^ to assess consistency of findings across different modeling approaches. We also assessed a mixed-effects model that controlled for within- and between-surgeon variability.

A 2-sided *P* value less than .05 was considered statistically significant. Data processing was conducted using SAS, version 9.4 (SAS Institute Inc), and statistical analyses were performed from April to August 2025 using Stata/MP, version 18 (StataCorp LLC).

## Results

### Surgeon and Patient Characteristics

This study included a total of 20 313 surgeons (mean [SD] age, 50.7 [10.2] years; 13.8% female, 86.2% male) who performed candidate procedures in 886 385 patients between January 1, 2011, and December 31, 2021. Among them, 5933 surgeons (29.2%) received a payment and 14 380 (70.8%) never received a payment (Table 1). Surgeons who received a payment performed 355 248 (40.1%) of the procedures included in the study. Surgeons who received payments were on average younger (mean [SD] age, 47.2 [9.2] vs 53.0 [10.1] years) and had fewer years of experience since medical school graduation (mean [SD], 20.1 [9.6] vs 26.0 [10.6] years). Of the patients, 56.3% were female and 43.7%, male; 1.2% were Asian, 8.6% were Black, 2.1% were Hispanic, 0.7% were North American Native, 86.0% were White, and 1.4% were other race and ethnicity. Mean (SD) patient age was 72.7 (10.9) years. At hospitals in the South, procedures were more likely to be performed by surgeons who received vs did not receive payments (45.1% vs 38.6%), and at hospitals in the Northeast, procedures by surgeons who had received payments were less likely (15.3% vs 20.4%). Patient characteristics, including age, sex, race and ethnicity, and comorbidity index, were similar between treated and control surgeons (Table 1).

### Increase in Industry Payments Over Time

**Figure 1.**
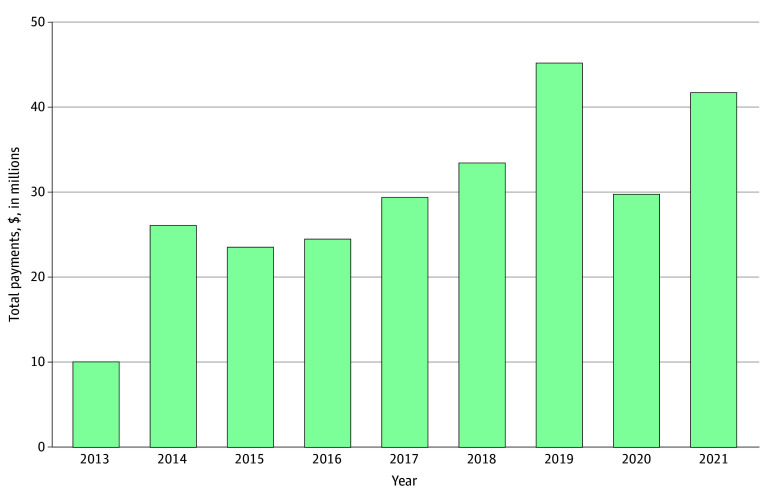
Bar Graph of Total Dollar Amount of Industry Payments From a Robotic Surgery Manufacturer to Physicians From 2013 to 2021 Payment amounts were aggregated annually.

The total approximate industry payments to physicians from the device company rose from $10 million in 2013 to $42 million in 2021, reaching a high of $45 million in 2019 (Figure 1). The total number of discrete payments also increased from 26 326 in 2013 to 61 495 in 2021.

### Association of Payment With Use of Robotic-Assisted Surgery

**Figure 2.**
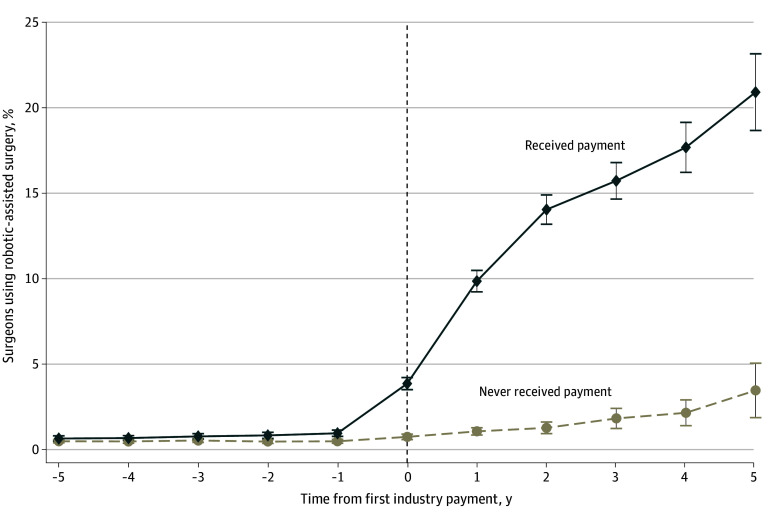
Line Graph of the Proportion of Surgeons Performing Robotic-Assisted Surgery Before and After Receipt of Industry Payment From a Robotic Surgery Manufacturer Estimates are derived from the difference-in-differences model, with robust SEs clustered at the surgeon level. Whiskers indicate 95% CIs. Year 0 represents the calendar year in which a surgeon first received an industry payment from the manufacturer. Surgeons who never received payments were randomly assigned a placebo event year within the study period to define relative time.

We did not observe evidence of differential pretreatment trends in the use of robotic-assisted surgery between treated and control surgeons, supporting the parallel trends assumption (eTable 2 in Supplement 1). Receipt of an industry payment was associated with a significant increase in the proportional use of robotic-assisted surgery, with a DID estimate of 9.9 percentage points (pp) (95% CI, 9.3-10.6 pp) (Table 2, Figure 2, and eFigure 1 in Supplement 1). Moreover, the proportional use of robotic-assisted surgery increased each year following payment. These results were consistent across each specific procedure as well. For example, receipt of an industry payment was associated with a significant increase in robotic-assisted surgery for bariatric surgery (11.7 pp; 95% CI, 9.4-13.9 pp) and ventral hernia repair (10.3 pp; 95% CI, 9.4-11.4 pp) (Table 2). Our estimates were also consistent across numerous different modeling specifications (eFigures 2-4 in Supplement 1). For the mixed-effects model sensitivity analysis, industry payment was associated with an increase in proportional use of robotic-assisted surgery of 7.4 pp (95% CI, 6.8-7.9 pp).

**Table 2.** DID Estimates of Industry Payment Association With Proportional Use of Robotic-Assisted Surgery by Procedure Type

Surgeon payment status^a^	Proportion using robotic-assisted surgery, % (95% CI)		Difference, pp (95% CI)	DID estimate, pp (95% CI)
	Before payment	After payment		
All surgeries				
Never received payment	0.6 (0.5-0.6)	1.4 (1.2 to 1.7)	0.9 (0.6 to 1.1)	9.9 (9.3-10.6)
Received payment	0.8 (0.7-1.0)	11.6 (11.0 to 12.3)	10.8 (10.2 to 11.4)	
Bariatric surgery				
Never received payment	0.4 (0.2-0.6)	1.0 (−0.2 to 2.2)	0.6 (−0.6 to 1.8)	11.7 (9.4-13.9)
Received payment	0.5 (0.2-0.9)	12.8 (10.9 to 14.7)	12.3 (10.4 to 14.2)	
Cholecystectomy				
Never received payment	0.3 (0.2-0.3)	0.7 (0.5 to 0.9)	0.5 (0.3 to 0.7)	7.4 (6.6-8.1)
Received payment	0.4 (0.3-0.4)	8.2 (7.5 to 8.9)	7.8 (7.1 to 8.6)	
Colectomy				
Never received payment	0.5 (0.4-0.7)	2.0 (1.4 to 2.6)	1.5 (1.0 to 2.0)	11.6 (10.5-12.7)
Received payment	0.8 (0.5-1.0)	13.9 (12.9 to 14.9)	13.1 (12.1 to 14.1)	
Ventral hernia repair				
Never received payment	1.4 (1.2-1.5)	2.2 (1.9 to 2.5)	0.9 (0.6 to 1.2)	10.3 (9.4-11.4)
Received payment	2.1 (1.8-2.4)	13.3 (12.3 to 14.3)	11.2 (10.3 to 12.2)	

Abbreviations: DID, difference in differences; pp, percentage points.

^a^ Surgeons who never received payments were randomly assigned a placebo event year within the study period to define relative time.

**Figure 3.**
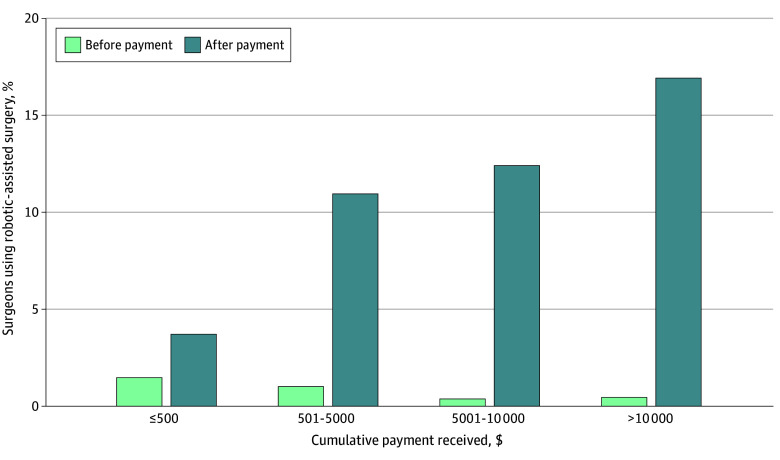
Bar Graph of the Mean Proportion of Surgeons Performing Robotic-Assisted Surgery Before and After Receiving Industry Payments From a Robotic Surgery Manufacturer, Grouped by Total Payment Amount

There was a consistent dose-dependent association between industry payments of higher dollar amounts and greater subsequent use of robotic-assisted surgery (Figure 3). For example, surgeons receiving $500 or less increased their proportional use of robotic-assisted surgery from a mean of 1.5% (95% CI, 1.4%-1.6%) to 3.7% (95% CI, 3.5%-3.9%), while surgeons receiving more than $10 000 increased use from 0.4% (95% CI, 0.4%-0.5%) to 17.0% (95% CI, 16.7%-17.3%).

## Discussion

This study of industry payments from a large robotic surgical device manufacturer to physicians had 3 key findings. First, the findings suggest payments are in general increasing each year, as robotic-assisted surgery continues to expand to new clinical domains. Second, among physicians performing general surgical operations, receipt of payment was associated with a substantial increase in the proportional use of robotic-assisted surgeries that persisted for years after the first payment. Third, we observed a consistent dose-dependent response to receipt of payment, whereby larger dollar amounts were associated with greater subsequent use of robotic-assisted surgery. Taken together, these findings indicate an association of industry payments with the rapid and widespread adoption of robotic-assisted surgery across the US.

Most evidence on the influence of industry payments on physician behavior or practice patterns focuses on the pharmaceutical industry, where the impact on patients is mixed. A 2023 study found that oncologists who received industry payments were nearly 20% more likely to prescribe nonrecommended or low-value drugs.^20^ On the other hand, industry payments can encourage the uptake of drugs that may help patients, as has been shown for certain cholesterol medications.^21^ There is also evidence of spillover effects, where industry payments from manufacturers of antiplatelet drugs not only increased prescribing of those medications but were also associated with an increase in physicians’ use of diagnostic and therapeutic percutaneous cardiac interventions.^22^ Most evidence from medical devices is consistent with what is observed for pharmaceuticals. Receipt of industry payments by physicians has been associated with increased use of devices across a range of clinical domains such as orthopedics and cardiology.^23,24^ In addition, there is some evidence that industry relationships are associated with publication of studies with more favorable outcomes related to the devices in question, including robotic-assisted surgery.^25,26^

That industry payments were associated with greater use of robotic-assisted surgery can be interpreted in several different ways. In simplest terms, this finding may reflect the realities of the medical device industry, where funded educational sessions and training (the nature of most payments) are required or strongly encouraged in order to obtain Food and Drug Administration clearance. In this context, the users of robotic surgical technologies are obligated to an industry relationship, and there may be real advantages. For example, device manufacturers ostensibly know the most about their technology and impart that experience to the end users. This deviates in 1 important way from relationships between physicians and the pharmaceutical industry: in the case of robotic-assisted surgery, the safety and effectiveness of the intervention is contingent on the proficiency of the surgeon, which may be out of the company’s control.

This study suggests that direct payments to surgeons are an effective means of changing practice patterns. Moreover, our data suggest that the association is not just indicative of a barrier to entry, as would be the case with educational expenses alone. Larger dollar amounts were associated with greater use of robotic surgical technologies. While this may reflect successful marketing on the part of a device manufacturer, it also may have an impact on the safety and value of surgical care across the US. For example, several recent studies have found massive uptake of robotic-assisted surgery despite an inferior safety profile in the case of cholecystectomy and worse long-term effectiveness in the case of ventral hernia repair.^13,15^ Therefore, while there may be true educational benefits of relationships between device manufacturers and physicians, the question is raised as to who is responsible for monitoring and ensuring the safe and effective diffusion of new technology. This may be especially true when the device in question also raises the costs of delivering the intervention, as has been shown for robotic-assisted surgery.^27^ Taken together, industry relationships may have compromised the value of care for some routine general surgical procedures.

### Limitations

These results should be interpreted within the context of several limitations. As with all DID analyses, our results rely on the assumption that absent industry payments, paid and unpaid surgeons would have followed similar trends in robotic-assisted surgery use. Estimates may also be sensitive to unmeasured time-varying confounding or contemporaneous changes in surgical practice patterns. Our analysis was restricted to common general surgical operations, and the results may not be generalizable to other specialties. We also focused on Medicare beneficiaries, which, depending on an individual surgeon’s case mix, could underestimate or overestimate the impact of industry payments on future use of robotic-assisted surgery. In addition, we examined payments to individual physicians rather than their institutions, where more tacit influence could shift practice patterns. Specifically, institutional priorities to grow robotic programs, rather than direct funds paid to individuals, may also drive surgeon behavior. However, most payments are made to physicians rather than institutions, and it would not be possible to understand the direct influence of institutional payments on discrete physicians. In addition, we focused on a study period in which robotic-assisted surgeries were increasing rapidly across the US. It is possible that the association between industry payment and subsequent use of this technology would be different in the future if utilization reached a steady state or even declined.

## Conclusions

In this cohort study of surgeons, receipt of industry payments from a large robotic surgical device company was associated with increased use of robotic-assisted surgery for common surgical procedures compared with no receipt of payments, with a significant dose-response association by payment amount. While relationships between industry and physicians serve important functions (eg, education about a new and more effective drug) that could benefit patients, they also warrant scrutiny in certain circumstances. With limited evidence of superior outcomes over existing, less-expensive approaches such as laparoscopic surgery, these data highlight how the activities of a robotic surgical device company could influence the value of care at a large scale.

## Notes

Supplement 1. **eTable 1.** *ICD-9* and *ICD-10* Procedure and Diagnosis Codes and *CPT* Codes for Cholecystectomy, Colectomy, Bariatric, and Hernia Repair Procedures **eTable 2.** Prepayment Slopes for Control and Treated Groups **eFigure 1.** Primary Model With Entropy Balancing **eFigure 2.** Unadjusted Model **eFigure 3.** FLEX Model **eFigure 4.** Callaway and Sant’Anna Method

Supplement 2.
Data Sharing Statement
