# Supplementary material for: Receipt of Industry Payments and Surgeons’ Adoption of Robotic-Assisted Surgery
Source: JAMA Netw Open. 2026 Mar 30;9(3):e263885. doi: 10.1001/jamanetworkopen.2026.3885 (PMC13036581; doi:10.1001/jamanetworkopen.2026.3885)
Supplement: Supplement 1. — eTable 1. ICD-9 and ICD-10 Procedure and Diagnosis Codes and CPT Codes for Cholecystectomy, Colectomy, Bariatric, and Hernia Repair Procedures eTable 2. Prepayment Slopes for Control and Treated Groups eFigure 1. Primary Model With Entropy Balancing eFigure 2. Unadjusted Model eFigure 3. FLEX Model eFigure 4. Callaway and Sant’Anna Method [file jamanetwopen-e263885-s001.pdf]

Supplementary Online Content

Loh WS, Norton EC, Thumma J, Dimick JB, Sheetz KH. Receipt of industry payments and surgeons' adoption of robotic-assisted surgery. *JAMA Netw Open*. 2026;9(3):e263885. doi:10.1001/jamanetworkopen.2026.3885

eTable 1. *ICD-9* and *ICD-10* Procedure and Diagnosis Codes and *CPT* Codes for Cholecystectomy, Colectomy, Bariatric, and Hernia Repair Procedures

eTable 2. Prepayment Slopes for Control and Treated Groups

eFigure 1. Primary Model With Entropy Balancing

eFigure 2. Unadjusted Model

eFigure 3. FLEX Model

eFigure 4. Callaway and Sant'Anna Method

This supplementary material has been provided by the authors to give readers additional information about their work.

© 2026 Loh WS et al. *JAMA Network Open*.

eTable 1: *ICD-9* and *ICD-10* procedure and diagnosis codes and *CPT* codes for cholecystectomy, colectomy, bariatric, and hernia repair procedures

Cholecystectomy	**ICD-9 Procedure Codes:** 5121, 5122, 5123, 5124<br><br>**ICD-10 Procedure Codes:** 0F540ZZ, 0F543ZZ, 0F544ZZ, 0FB40ZZ, 0FB43ZZ, 0FB44ZZ, 0FT40ZZ, 0FT44ZZ<br><br>**CPT Codes:** 49310, 56340, 56342, 47562, 47564, 47600, 47610, 47612, 476
Colectomy	**ICD-9 Procedure Codes:**<br>1731-1739, 4571, 4572, 4573, 4574, 4575, 4576, 4579, 4581, 4582, 4583<br><br>**ICD-10 Procedure Codes:** 0DBE0ZZ, 0DBE3ZZ, 0DBE4ZZ, 0DBE7ZZ, 0DBF0ZZ, 0DBF3ZZ, 0DBF4ZZ, 0DBF7ZZ, 0DBG0ZZ, 0DBG3ZZ, 0DBG4ZZ, 0DBG7ZZ, 0DBH0ZZ, 0DBH3ZZ, 0DBH4ZZ, 0DBH7ZZ, 0DBK0ZZ, 0DBK3ZZ, 0DBK4ZZ, 0DBK7ZZ, 0DBL0ZZ, 0DBL3ZZ, 0DBL4ZZ, 0DBL7ZZ, 0DBM0ZZ, 0DBM3ZZ, 0DBM4ZZ, 0DBM7ZZ, 0DBN0ZZ, 0DBN3ZZ, 0DBN4ZZ, 0DBN7ZZ, 0DTE0ZZ, 0DTE4ZZ, 0DTE7ZZ, 0DTF0ZZ, 0DTF4ZZ, 0DTF7ZZ, 0DTG0ZZ, 0DTG4ZZ, 0DTG7ZZ, 0DTH0ZZ, 0DTH4ZZ, 0DTH7ZZ, 0DTK0ZZ, 0DTK4ZZ, 0DTK7ZZ, 0DTL0ZZ, 0DTL4ZZ, 0DTL7ZZ, 0DTM0ZZ, 0DTM4ZZ, 0DTM7ZZ, 0DTN0ZZ, 0DTN4ZZ, 0DTN7ZZ<br><br>**CPT Codes:** 44140, 44141, 44143, 44144, 44145, 44146, 44147, 44150, 44151, 44155, 44156, 44157, 44158, 44160, 44204, 44205, 44206, 44207, 44208, 44210, 44211, 44212
Bariatric surgery	**ICD-9 Diagnosis Codes:** 2780, 27801 ,27800<br><br>**ICD-9 Procedure Codes**: 443, 4431, 4438, 4439, 4389, 4468, 4495, 4496, 4499, 445, 4382<br><br>**ICD-10 Diagnosis Codes:** E6609, E661, E668, E669, E6601, E663<br><br>**ICD-10 Procedure Codes:** 0DB63Z3, 0DB63ZZ, 0DB64Z3, 0DB67Z3, 0DB67ZZ, 0DB68Z3, 0DH60DZ, 0DH63DZ, 0DH64DZ ,0DH67DZ, 0DH68DZ, 0DL60CZ, 0DL60DZ, 0DL60ZZ, 0DL63CZ, 0DL63DZ, 0DL63ZZ, 0DL64CZ, 0DL64DZ, 0DL64ZZ, 0DL67DZ, 0DL67ZZ, 0DL68DZ, 0DL70CZ, 0DL70DZ, 0DL70ZZ, 0DL73CZ, 0DL73DZ, 0DL73ZZ, 0DL74CZ,0DL74DZ, 0DL74ZZ, 0DL77DZ, 0DL77ZZ, 0DL78DZ, 0DQ60ZZ, 0DQ63ZZ, 0DQ64ZZ, 0DQ67ZZ, 0DV64CZ, 0DW643Z, 0DW64CZ

© 2026 Loh WS et al. *JAMA Network Open*.

	**CPT Codes:** 43770, 43771, 43772, 43773, 43774, 43775, 43847, 43845, 43644, 43645, 43842, 43843, 43844, 43845, 43846, 43847, 43848, S2083
Hernia Repair	**ICD-9 Diagnosis Codes:** 551.1, 551.2, 551.20, 551.21, 551.29, 552.1, 552.2, 552.20, 552.21, 552.29, 553.1, 553.2, 553.20, 553.21, 553.29<br><br>**ICD-9 Procedure Codes:** 53.4, 53.41, 53.42, 53.43, 53.49, 53.5, 53.51, 53.59, 53.6, 53.61, 53.62, 53.63, 53.69<br><br>**ICD-10 Diagnosis Codes:** K42, K42.0, K42.1, K42.9, K43, K43.0, K43.1, K43.2, K43.6, K43.7, K43.9<br><br>**ICD-10 Procedure Codes:** 0WQF0ZZ, 0WQF3ZZ, 0WQF4ZZ, 0WQFXZZ, 0WMF0ZZ, 0WUF07Z, 0WUF0JZ, 0WUF0KZ, 0WUF47Z, 0WUF4JZ, 0WUF4KZ<br><br>**CPT Codes:** 49560, 49561, 49565, 49566, 49570, 49572, 49585, 49587, 49652, 49653, 49654, 49655, 49656, 49657, S2900
Robotic-assisted identifier codes	**ICD-9 Procedure Codes:** 1741, 1742, 1743, 1744, 1744, 1746, 1747,1748, 1749<br><br>**ICD-10 Procedure codes:** 8E0W0CZ, 8E0W3CZ, 8E0W4CZ, 8E0W4CZ, 8E0W7CZ,<br>8E0W8CZ, 8E0WXCZ<br><br>**CPT code:** S2900

© 2026 Loh WS et al. *JAMA Network Open*.

eTable 2: Prepayment slopes for control and treated groups.

Surgical Procedure	Surgeon Group	Pre-Payment Slope (95% CI)
All Surgeries	Never Received Payment	-0.003 (-0.04–0.03)
	Received Payment	0.08 (0.028–0.13)
Bariatric Surgery	Never Received Payment	0.03 (-0.1–0.2)
	Received Payment	-0.005 (-0.1–0.1)
Cholecystectomy	Never Received Payment	-0.04 (-0.07– -0.01)
	Received Payment	0.04 (0.003-0.08)
Colectomy	Never Received Payment	-0.01 (-0.08–0.05)
	Received Payment	0.14 (0.04–0.2)
Hernia Repair	Never Received Payment	0.14 (0.05–0.2)
	Received Payment	0.13 (-0.01–0.3)

© 2026 Loh WS et al. *JAMA Network Open*.

eFigure 1 – Primary model with entropy balancing

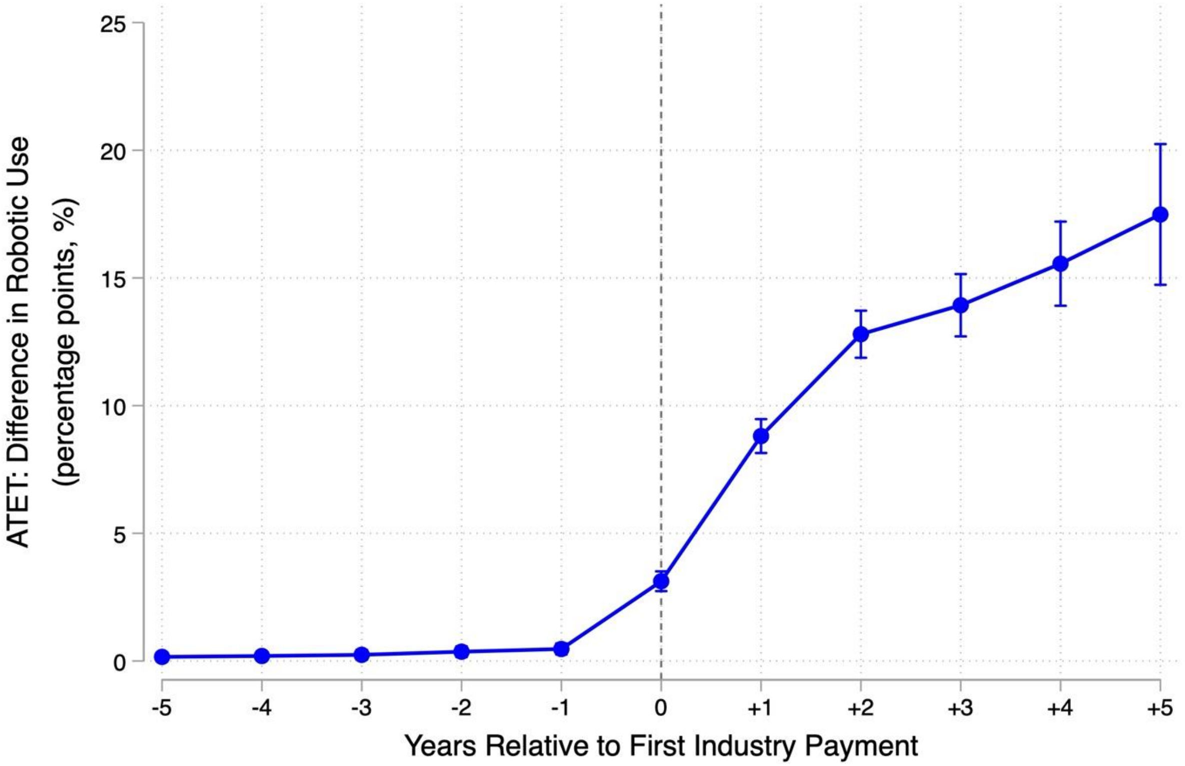

This figure shows the difference in proportional use of robotic-assisted surgery (Average Treatment Effect on the Treated, ATET) between surgeons who received a payment from Intuitive Surgical, Inc. and those who did not, by year relative to the first payment. Estimates were derived from a weighted DID model with robust standard errors clustered at the surgeon level. Models were adjusted for patient demographics (age, sex, race), comorbidity burden (sum of 29 Elixhauser comorbidities, ranging from 0 to 29), hospital characteristics (teaching status, ownership type, bed size category, urban location, census region), surgeon characteristics (age, sex, years in experience), and calendar-year fixed effects using entropy balance weights.

© 2026 Loh WS et al. *JAMA Network Open*.

eFigure 2 – Unadjusted model

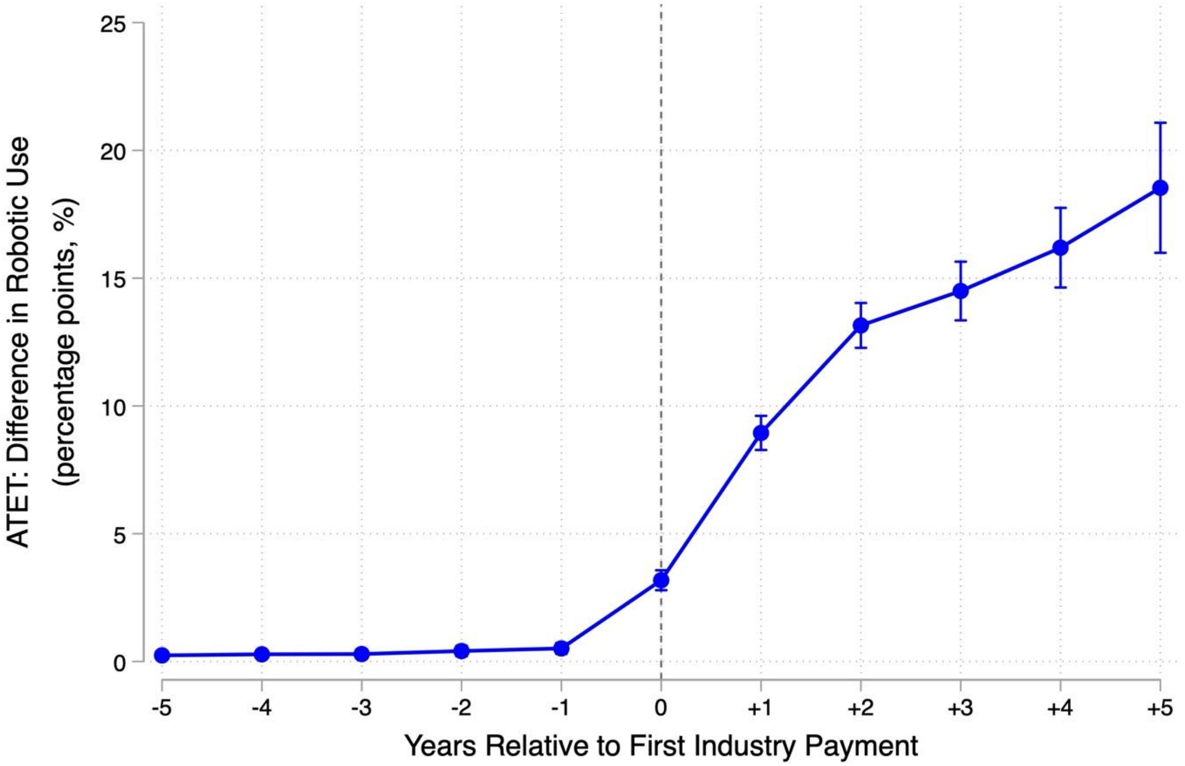

This figure shows the difference in robotic-assisted surgery use (Average Treatment Effect on the Treated, ATET) between surgeons who received a payment from Intuitive Surgical, Inc. and those who did not, by year relative to the first payment. Estimates were derived from an unadjusted DID model.

© 2026 Loh WS et al. *JAMA Network Open*.

eFigure 3 – FLEX model

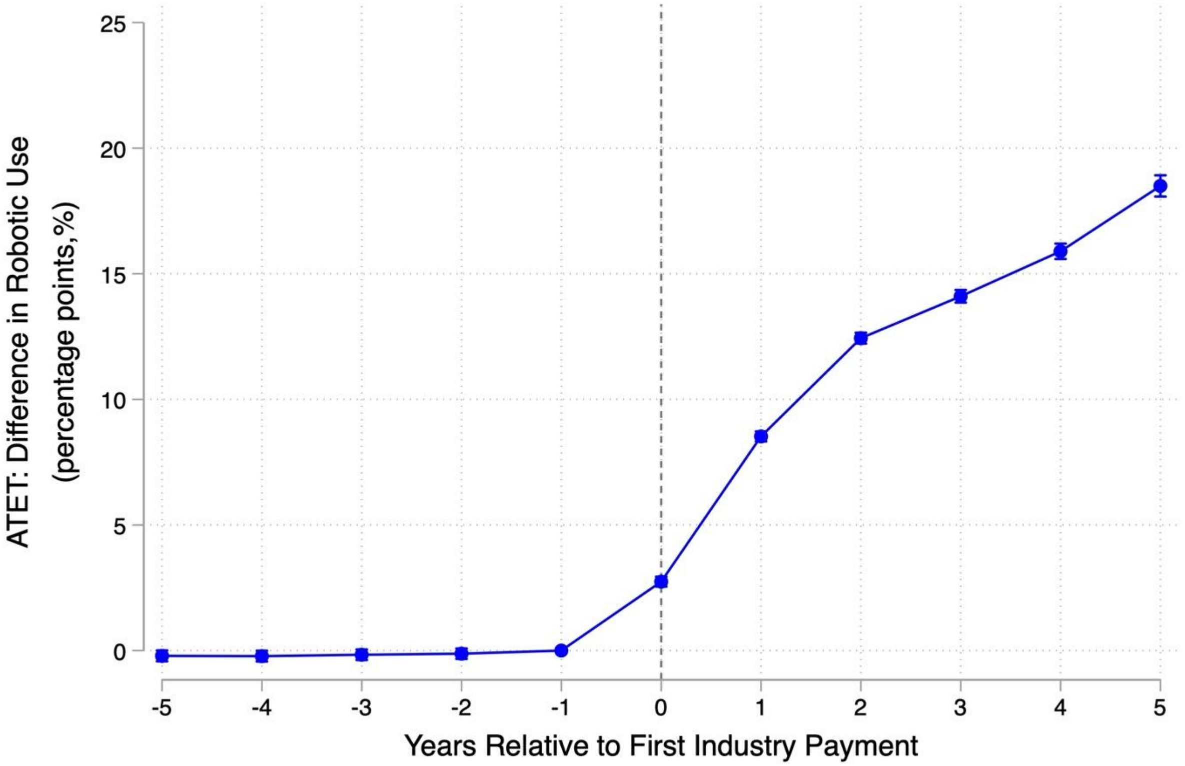

This figure shows the difference in robotic-assisted surgery use (Average Treatment Effect on the Treated, ATET) between surgeons who received a payment from Intuitive Surgical, Inc. and those who did not, by year relative to the first payment. Estimates are derived from flexible linear model estimated by OLS with covariates (FLEX) introduced by Deb et al. 2024. FLEX is used to estimate treatment effects in difference-in-differences study in which the treatment start time staggered over time and treatment effects are heterogenous by group, time, and covariates, and when the data are repeated cross-sections.

© 2026 Loh WS et al. *JAMA Network Open*.

eFigure 4 - Callaway and Sant'Anna method

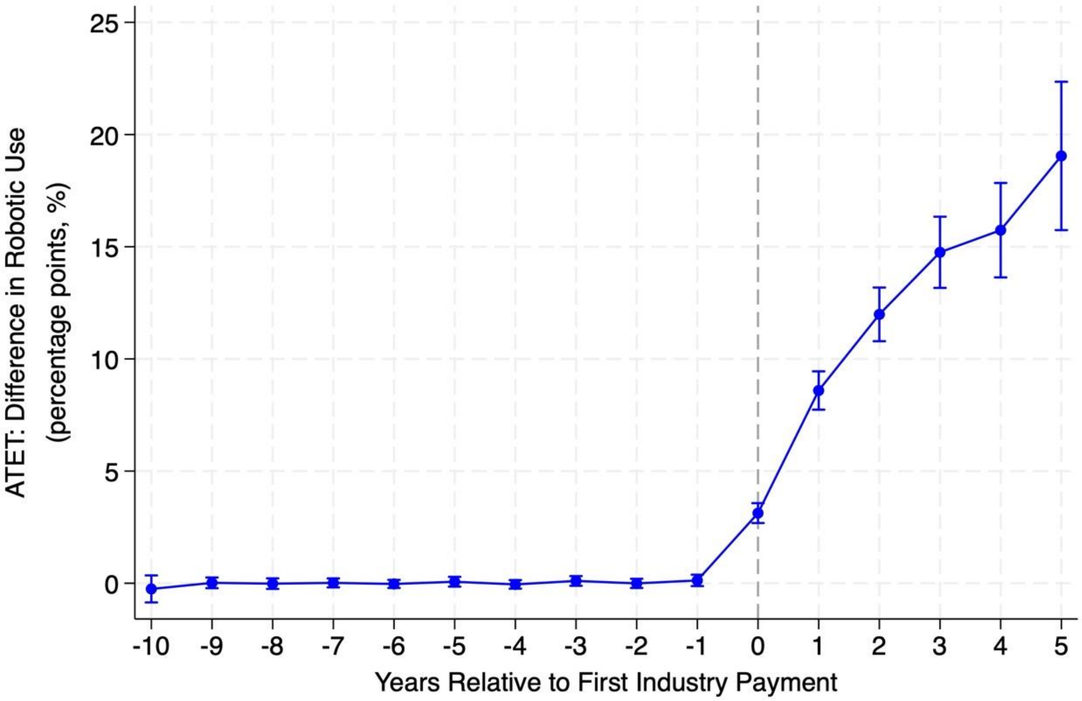

This figure shows the difference in robotic-assisted surgery use (Average Treatment Effect on the Treated, ATET) between surgeons who received a payment from Intuitive Surgical, Inc. and those who did not, by year relative to the first payment. Estimates were derived from Callaway and Sant'Anna method by first industry payment year, which extends traditional difference-in-differences methods by flexibly estimating variation in treatment effects across cohorts and over time.

© 2026 Loh WS et al. *JAMA Network Open*.
