# Supplementary material for: Receipt of Industry Payments and Surgeons’ Adoption of Robotic-Assisted Surgery
Source: JAMA Netw Open. 2026 Mar 30;9(3):e263885. doi: 10.1001/jamanetworkopen.2026.3885 (PMC13036581; doi:10.1001/jamanetworkopen.2026.3885)
Supplement: Supplement 2. — Data Sharing Statement [file jamanetwopen-e263885-s002.pdf]

Data Sharing Statement

San Loh. Receipt of Industry Payments and Surgeons’ Adoption of Robotic-Assisted Surgery. *JAMA Netw Open*. Published March 30, 2026. doi:10.1001/jamanetworkopen.2026.3885

Data
Data available: No

Additional Information
Explanation for why data not available: Medicare does not permit sharing of data. The data on industry payments is publicly available.
